# Endogenous opioids contribute to insensitivity to pain in humans and mice lacking sodium channel Na_v_1.7

**DOI:** 10.1038/ncomms9967

**Published:** 2015-12-04

**Authors:** Michael S. Minett, Vanessa Pereira, Shafaq Sikandar, Ayako Matsuyama, Stéphane Lolignier, Alexandros H. Kanellopoulos, Flavia Mancini, Gian D. Iannetti, Yury D. Bogdanov, Sonia Santana-Varela, Queensta Millet, Giorgios Baskozos, Raymond MacAllister, James J. Cox, Jing Zhao, John N. Wood

**Affiliations:** 1 Molecular Nociception Group, WIBR, University College London, Gower Street, London WC1E 6BT, UK; 2 Department of Neuroscience, Physiology and Pharmacology, University College London, Gower Street, London WC1E 6BT, UK; 3 Institute of Structural and Molecular Biology, UCL, London WC1E 6BT, UK; 4 Department of Medicine, UCL, London WC1E 6BT, UK

## Abstract

Loss-of-function mutations in the *SCN9A* gene encoding voltage-gated sodium channel Na_v_1.7 cause congenital insensitivity to pain in humans and mice. Surprisingly, many potent selective antagonists of Na_v_1.7 are weak analgesics. We investigated whether Na_v_1.7, as well as contributing to electrical signalling, may have additional functions. Here we report that Na_v_1.7 deletion has profound effects on gene expression, leading to an upregulation of enkephalin precursor *Penk* mRNA and met-enkephalin protein in sensory neurons. In contrast, Na_v_1.8-null mutant sensory neurons show no upregulated *Penk* mRNA expression. Application of the opioid antagonist naloxone potentiates noxious peripheral input into the spinal cord and dramatically reduces analgesia in both female and male Na_v_1.7-null mutant mice, as well as in a human Na_v_1.7-null mutant. These data suggest that Na_v_1.7 channel blockers alone may not replicate the analgesic phenotype of null mutant humans and mice, but may be potentiated with exogenous opioids.

## Abstract (web summary)

Na_v_1.7 channels are known to regulate pain perception in humans and mice. Here, the authors provide evidence that Na_v_1.7 deletion leads to transcriptional upregulation of opioid peptides in sensory neurons, and that treatment with the opioid blocker naloxone helps reverse analgesia in mice and human Na_v_1.7 nulls.

## Graphical abstract

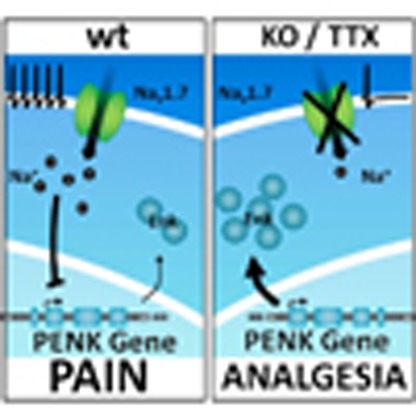

The problem of pain continues to grow as populations age; about one in five are victims, with 7% suffering debilitating, poorly treated chronic pain^1^. Despite this vast clinical problem, little progress has been made in developing new therapeutic agents. Sensory neurons that respond to tissue damage and drive central pain pathways have been a focus of analgesic drug development, because nerve block relieves most pains, and a unique repertoire of sodium channels are found in peripheral sensory neurons^2^,^3^. *SCN9A* encodes a voltage-gated sodium channel, Na_v_1.7, which is found in the peripheral sensory and sympathetic neurons, as well as in olfactory neurons, the hypothalamus and some non-neural tissue such as the pancreas^2^,^3^,^4^,^5^. Deletion of Na_v_1.7 in sensory and sympathetic neurons of mice leads to a pain-free congenital insensitivity to pain (CIP) phenotype similar to that described in humans^5^,^6^,^7^,^8^. Apart from anosmia, human and mouse Na_v_1.7-null mutants are apparently normal, suggesting that this channel is an excellent analgesic drug target for acute, inflammatory and neuropathic pain. One caveat to this conclusion is that loss of Na_v_1.7 has also been associated with peripheral neuropathy in humans and this could potentially contribute to peripheral analgesia^3^,^9^. However, in Na_v_1.7-null mutant mouse models that recapitulate the human CIP phenotype, there is no evidence for any sensory neuronal cell loss^10^. Loss of Na_v_1.7 does result in some mouse sensory neurons becoming electrically silenced at normal resting potentials, consistent with a role for Na_v_1.7 in action potential propagation in nociceptive neurons^11^. Nerve block through the use of broad-spectrum sodium channel antagonists as local anaesthetics is a very effective way to treat pain, but inhibition of innocuous sensation makes this approach impractical for most indications. However, Na_v_1.7 antagonists are not local anaesthetics and, if selective, should show none of the side effects such as cardiotoxicity that are associated with broad-spectrum sodium channel blockers. Despite considerable efforts, no evidence for the dramatic analgesia found in Na_v_1.7-null mutants has been obtained for compounds such as Protoxin II, which selectively and potently target Na_v_1.7, whilst neutralizing monoclonal antibodies evoke only partial analgesia that lasts for <24 h (refs 12, 13). Here we describe experiments that help explain this apparent anomaly. We report that Na_v_1.7 deletion leads to increased transcription of *Penk* messenger RNA and higher levels of enkephalins in sensory neurons. The analgesia associated with loss of Na_v_1.7 in both mice and humans is substantially reversed by the opioid antagonist naloxone. Thus, Na_v_1.7 deletion increases endogenous opioid-dependent analgesia as well as diminishing peripheral nociceptive drive in pain states.

## Results

Many proteins have several, quite distinct functions. For example, some chromatin proteins such as high-mobility group box 1 protein are also extracellular signalling molecules^14^, whereas the β4-subunit of voltage-gated calcium channels has been shown to have a role as a transcription factor^15^. We wondered whether Na_v_1.7 had an additional role to that of propagating action potentials. We therefore analysed the patterns of gene expression in the sensory neurons of dorsal root ganglia (DRG) from Na_v_1.7, Na_v_1.8 and Na_v_1.9 knockout (KO) mice^16^,^17^,^18^. Whole dorsal root ganglia were included in the analysis, but as the sodium channels are neuronal proteins, alterations in gene expression can be ascribed to changes in the sensory neuron transcriptome. The three channels are all expressed at high levels in peripheral damage-sensing neurons and have been linked to human loss- or gain-of-function pain conditions^2^,^3^. Na_v_1.9 seems to play a role in setting thresholds of activation, whereas Na_v_1.7, which is expressed in most DRG neurons, transmits action potentials that are generated by noxious stimuli that depolarize the neurons^18^. Na_v_1.8, which is specifically expressed in the same subset of sensory neurons that express Na_v_1.9, is responsible for much of the nociceptive electrical input into the central nervous system and is activated at more negative potentials than Na_v_1.7. Na_v_1.8 seems to be particularly important for nociceptive signalling at low temperatures^2^,^17^. Interestingly, Na_v_1.7 plays an essential role in neurotransmitter release, whereas Na_v_1.8 does not^2^,^5^. Thus, some functions of these sodium channels, both associated with action potential propagation, are distinct, and this may reflect different biophysical properties, subcellular locations or association with different protein complexes.

**Figure 1:**
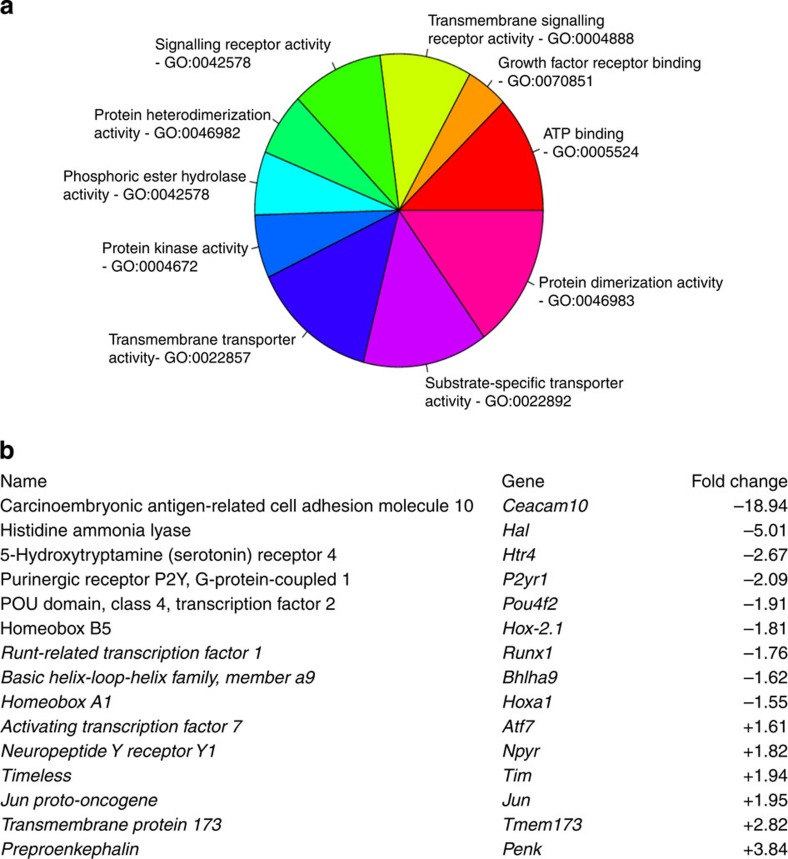
Deletion of Na_v_1.7 leads to altered gene expression in DRG neurons. (**a**) The pie chart shows the number of significantly differentially expressed (DE) genes (analysis of variance *P*-value<0.01) between Na_v_1.7 KO mice (*n*=3) and wild-type littermates (*n*=3), annotated with the top enriched Gene Ontology (GO) terms regarding the Biological Process (BP). For the enrichments analysis, we used methods based both on gene counts, namely the classical Fisher test, and gene ranks and scores, namely the Kolmogorov–Smirnov-type tests. (**b**) Example list of the fold change of differentially expressed genes in sensory neurons from Na_v_1.7 KO mice. Complete data are available at GEO and ArrayExpress, accession code GSE61373.

Despite the significant role of all three sensory neuron-associated sodium channels in peripheral pain pathways, we found a much more dramatic alteration in gene expression in DRG from mice in which Na_v_1.7 has been conditionally deleted from all sensory neurons using Advillin-Cre^7^ (194 genes>1.5 fold dysregulated; Fig. 1a) than in global Na_v_1.8 (17 genes)- or Na_v_1.9 (64 genes)-null mutant mice (Gene Expression Omnibus accession number GSE61373). In Na_v_1.7 conditional KO mice, a number of transcription factors associated with sensory neuron subsets were dysregulated including *Brn3B*, *Runx1* and *HoxB5* (Fig. 1b). Most strikingly, the levels of *Penk* mRNA, the precursor of Leu- and Met-enkephalin^19^, were upregulated in Na_v_1.7, but not in Na_v_1.8- or Na_v_1.9-null mutant mice (Fig. 1b and Supplementary Tables 1 and 2), whereas the transcript of the little characterized gene *Ceacam10* (ref. 20) was dramatically downregulated in both Na_v_1.7- and Na_v_1.8-null mice (Fig. 1b and Supplementary Table 1). This raised the possibility that enkephalin-mediated analgesia could contribute to the CIP phenotype of Na_v_1.7-null mutant mice and humans.

**Figure 2:**
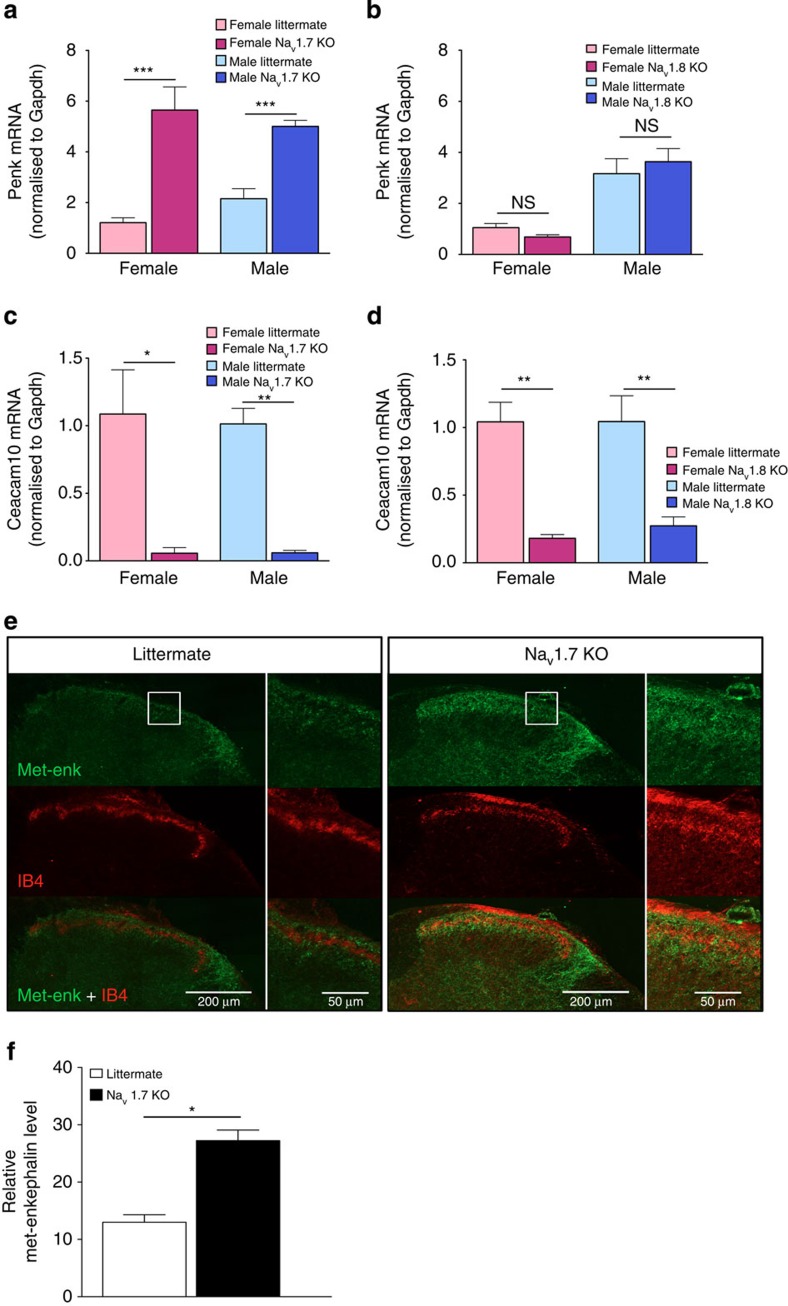
*Scn9a* deletion increases *Penk* mRNA expression in DRG neurons. Reverse transcriptase–PCR (RT–PCR) analysis of *Penk* and *Ceacam10* mRNA expression in DRG relative to *Gapdh* mRNA levels in wild-type littermates and Na_v_1.7 KO mice. (**a**) Increased expression of *Penk* mRNA was observed in both female Na_v_1.7 KOs (light pink column, *n*=3) compared with female littermates (dark pink column, *n*=3), as well as male Na_v_1.7 KOs (light blue column, *n*=3) compared with male littermates (dark blue column, *n*=3). No difference was observed between wild-type females and wild-type males. (**b**) *Penk* mRNA expression was not altered in Na_v_1.8 KOs compared with littermates (female Na_v_1.8 KOs: light pink column, *n*=3; female littermates: dark pink column, *n*=3; male Na_v_1.8 KOs: light blue column, *n*=3; male littermates: dark blue column, *n*=3). (**c**) *Ceacam10* mRNA was downregulated in both female Na_v_1.7 KOs (light pink column, *n*=3) compared with female littermates (dark pink column, *n*=3) and male Na_v_1.7 KOs (light blue column, *n*=3) compared with male littermates (dark blue column, *n*=3). (**d**) Downregulation of *Ceacam10* mRNA was also observed in Nav1.8 KOs compared with littermates (female Na_v_1.8 KOs: light pink column, *n*=3; female littermates: dark pink column, *n*=3; male Na_v_1.8 KOs: light blue column, *n*=3; male littermates: dark blue column, *n*=3). All results are shown as a fold change from wild-type littermate. (**e**) L5–L6 spinal cord sections were double-labelled with met-enkephalin (Green) and IB-4 (Red). Right panel insert shows higher magnification of lamina I and II within the dorsal horn. (**f**) Quantification of immunostaining signal shows less Met-enkephalin immunoreactivity in littermate dorsal horns (*n*=3) compared with the Na_v_1.7 KO dorsal horn (*n*=3). Data are shown as mean±s.e.m. All RT–PCR data are expressed as mean±s.e.m. with significance indicated by *P<0.05, **P<0.01 and ***P<0.001 (one-way analysis of variance with Bonferroni post test (**a**,**b**)). Immunoreactivity signal is expressed as mean±s.e.m. with significance indicated by *P<0.05 (Student's *t*-test).

We confirmed the microarray data on *Penk* expression using quantitative PCR (qPCR). We found that *Penk* mRNA was upregulated in both female and male Na_v_1.7-null mutant mice (Fig. 2a) but not in Na_v_1.8-null mutant DRG (Fig. 2b), whereas *Ceacam10* mRNA was downregulated in both null mutant mouse lines (Fig. 2c,d). The overexpression of *Penk* mRNA was replicated at the protein level in the central terminals of primary afferents. Met-enkephalin immunoreactivity was strongly enhanced in Na_v_1.7-null mutants compared with littermate control mice (Fig. 2e). The increase in immunoreactive Met-enkephalin was more than twofold (Fig. 2f).

**Figure 3:**
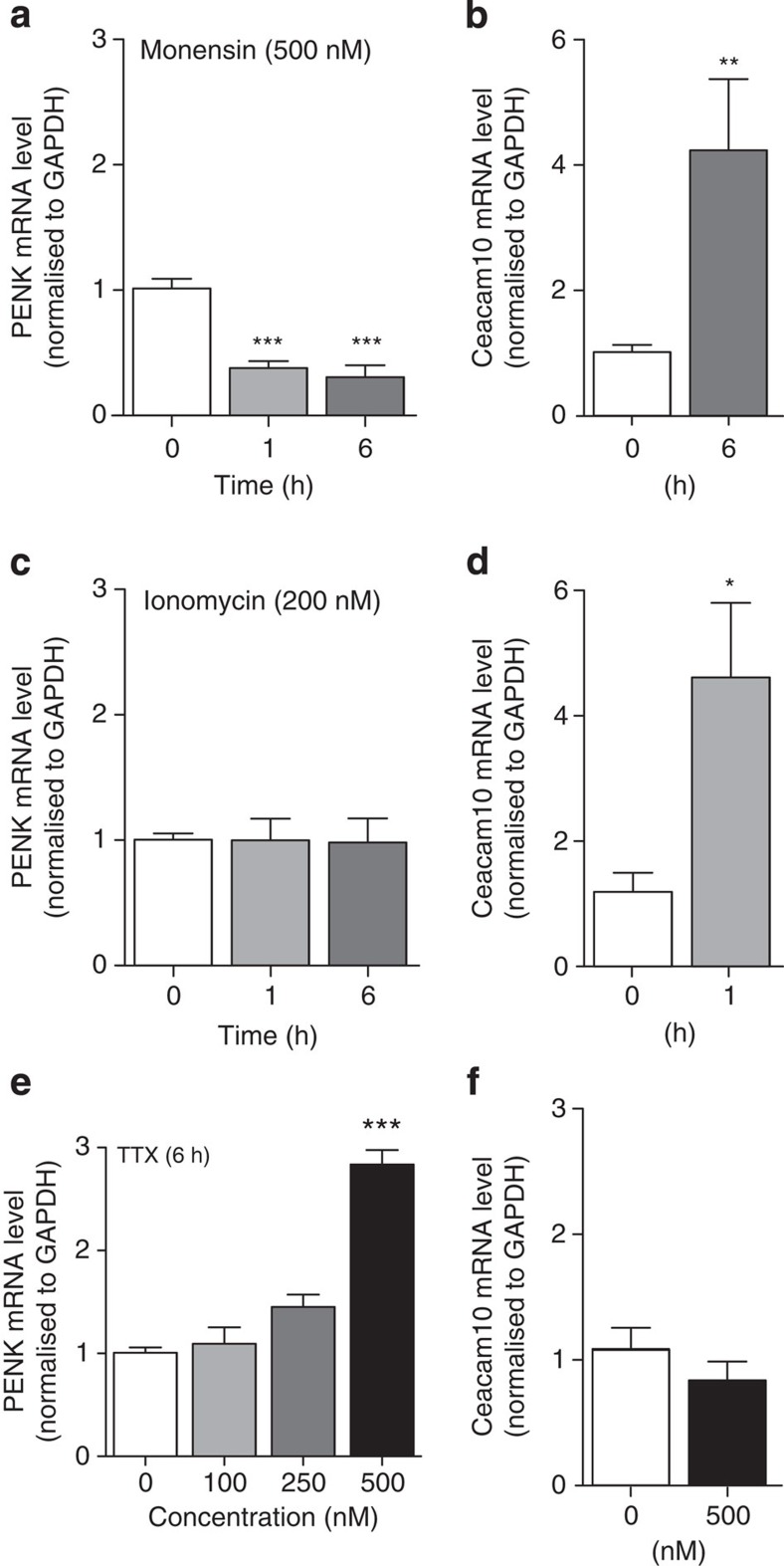
Intracellular sodium concentration regulates *Penk* expression. Time-course reverse transcriptase–PCR (RT–PCR) experiments were performed to quantify expression of *Penk* and *Ceacam10* mRNA in cultured DRG neurons in response to intracellular changes in cation concentration (*n*=6 per group). (**a**) Monensin-mediated intracellular sodium rise decreased *Penk* mRNA expression in DRG neurons. (**b**) Under the same conditions, *Ceacam10* mRNA level was upregulated. (**c**) *Penk* expression was not altered by intracellular calcium rise induced by ionomycin, (**d**) whereas *Ceacam10* mRNA was significantly upregulated after 1 h of incubation with the calcium ionophore. (**e**) Exposure (6 h) to the voltage-gated sodium channel blocker TTX induces a dose-dependent upregulation of *Penk* expression. (**f**) At the highest TTX concentration, 500 nM, *Ceacam10* expression level was not altered. All RT–PCR data are expressed as mean±s.e.m. with significance indicated by *P<0.05, **P<0.01 and ***P<0.001 (one-way analysis of variance with Dunnett's post test or Student's *t*-test).

How does the loss of a voltage-gated sodium channel alter levels of transcriptional activity? The activity of Na_v_1.7 as a major sodium selective ion channel in sensory neurons could be linked to altered gene expression through effects on second messengers. Changes in sodium levels could have an indirect effect through altered intracellular calcium levels driven by pumps or the sodium/calcium exchangers found in sensory neurons^21^. However, evidence of a direct role for sodium as a transcriptional regulator has come from studies of atrial myocytes and kidney medullary cells where intracellular sodium acting through a salt-dependent kinase can influence gene expression through regulation of the transcription factor nuclear factor of activated T-cells 5 (NFAT5; refs 22, 23). A doubling of intracellular sodium levels in response to carbachol has been described in early studies of sympathetic neurons^24^. We examined the consequences of altering intracellular sodium and calcium levels on *Penk* and *Ceacam10* mRNA expression. We tested the effects of monensin, a sodium ionophore that can elevate intracellular sodium levels, and found that monensin (0.5 μM) could downregulate the expression of *Penk* mRNA with no effects on the housekeeping gene *Gapdh* in DRG neurons derived from wild-type mice (Fig. 3a), whereas *Ceacam10* expression was enhanced a few-fold by monensin treatment (Fig. 3b). This suggests a potential role for sodium as a second messenger in sensory neurons in terms of regulation of gene expression.

Calcium is the cation more usually linked to second messenger activity through interaction with a range of enzymes. Altered levels of intracellular sodium ions may result in changes in intracellular calcium through effects on pumps and exchangers^21^. Moreover, at high concentrations, monensin is able to increase intracellular calcium levels in some cells^25^. We used the calcium ionophore ionomycin to explore the potential link between altered calcium levels and gene expression. Ionomycin (0.2 μM), unlike monensin, had no effect on *Penk* mRNA levels measured using qPCR in DRG neurons (Fig. 3c). Interestingly, the increase in intracellular calcium caused by ionomycin led, as with monensin treatment, to enhanced expression of *Ceacam10* mRNA, showing that calcium-mediated second messenger regulatory events are at play in sensory neurons in these experiments (Fig. 3d). However, as the increase in levels of intracellular calcium had no effect on *Penk* mRNA levels, sodium rather than calcium seems to be a key second messenger in the regulation of opioid peptide expression. To explore the mechanism of *Penk* mRNA regulation further, we used the sodium channel-specific pore blocker tetrodotoxin (TTX) to explore changes in gene expression^26^. The half-maximal inhibitory concentration (IC_50_) for TTX is 30 nM for Na_v_1.7; however, Na_v_1.8 is defined as TTX insensitive with an IC_50_ of 60 μM (refs 16, 17). By using TTX at concentrations up to 500 nM, we could completely block the activity of Na_v_1.7 and other TTX-sensitive sodium channels all known to be present in DRG, whereas Na_v_1.8 would retain activity. Interestingly, channel block with TTX leads to an enhanced expression of *Penk*, slightly lower in magnitude to that caused by the deletion of Na_v_1.7 (Fig. 3e), but is without effect on *Ceacam10* expression (Fig. 3f). This supports the notion that sodium ingress through Na_v_1.7 channels downregulates *Penk* mRNA, although the regulation of *Ceacam10* expression requires further investigation.

**Figure 4:**
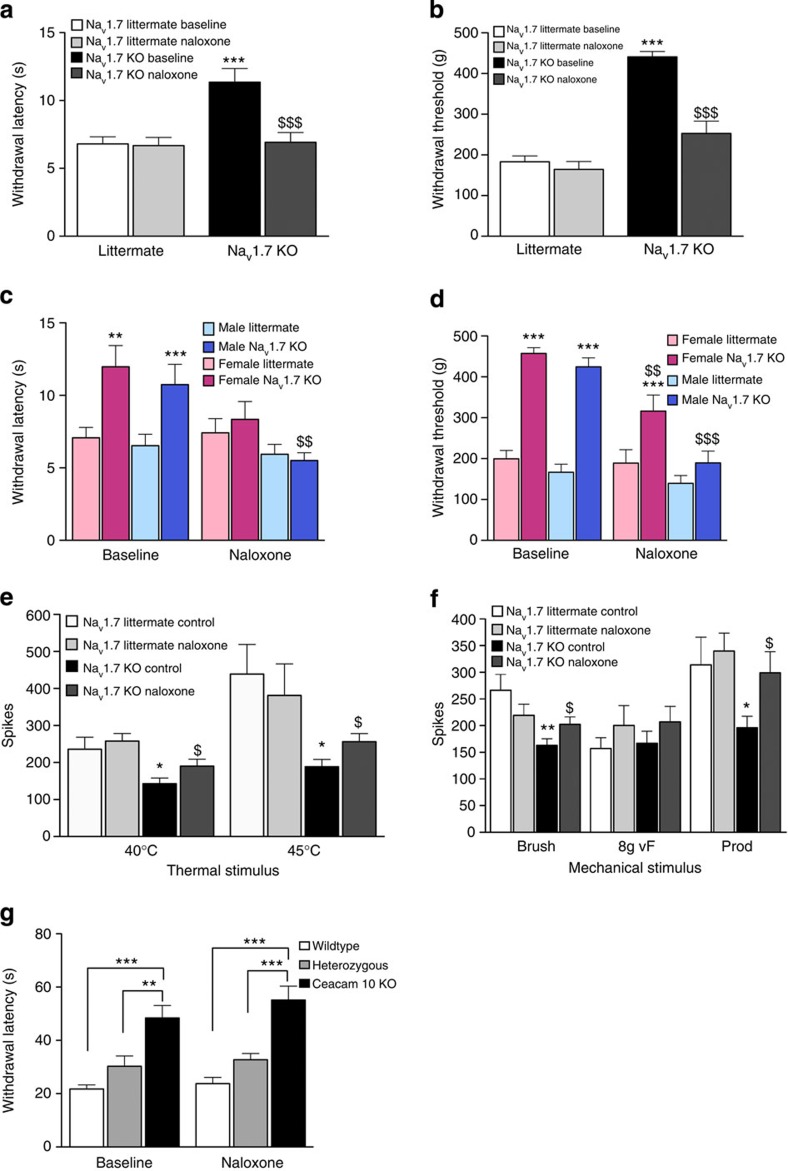
Mechanical and thermal sensory deficits in Na_v_1.7-null mice are reversed with the opioid antagonist naloxone. Hargreaves ((**a**,**c**) *n*=6 female and *n*=6 male per genotype) and Randall-Selitto ((**b**,**d**) *n*=3 female and *n*=3 male per genotype) pain behavioural tests show higher pain thresholds of Na_v_1.7 KO mice compared with littermates. Systemic naloxone reduces thermal and pressure pain thresholds of male and female Na_v_1.7 KO mice but has no effect on littermates. Thermally evoked (**e**) and mechanically evoked (**f**) firing of deep dorsal horn WDR neurons is lower in Na_v_1.7 KO mice (*n*=17 neurons) compared with littermate controls (*n*=24 neurons). Systemic naloxone sensitizes evoked neuronal firing to both low and high threshold thermal stimulation, as well as high threshold and dynamic (brush) mechanical stimulation, with no effect on control littermates. (**g**) Ceacam10 KO and heterozygous KO mice show clear thermal analgesia in the Hargreaves test and this is unaffected by naloxone administration (*n*=6 per genotype). Data are expressed as mean±s.e.m. *P<0.05, **P<0.01 and ***P<0.001 significance levels indicate differences between KOs and littermates using a Student's *t*-test and $*P*<0.05, $$*P*<0.01 and $$$ *P*<0.001 indicate differences following naloxone administration using a paired *t*-test.

The present data demonstrate that enkephalins are upregulated in Na_v_1.7-null mutant mice. We therefore used the opioid antagonist naloxone to examine the role of endogenous opioids in pain behaviour in Na_v_1.7-null mutant mice^27^. These mice were generated by means of an Advillin-Cre mouse that only expresses recombinase activity in sensory neurons^7^. Recent RNA sequencing data highlights the significance of the μ-opioid receptor in nociceptive Na_v_1.8-expressing neurons that do not contain mRNA for κ- or δ-opioid receptors^28^. The target of naloxone on sensory neurons is thus likely to be the μ-opioid receptor. We used measures of both thermal and mechanical pain^29^. When naloxone was administered to floxed Na_v_1.7–Advillin-Cre-null mutant mice, there was a dramatic reversal of analgesia and restoration of thermal and mechanical pain thresholds in the Hargreaves and Randall-Selitto tests respectively, to levels close to that found in littermate controls (Fig. 4a,b). The dose of naloxone used had no effect on pain thresholds of littermate controls. Both female and male Na_v_1.7-null mutants showed substantial reversal of analgesia with naloxone (Fig. 4c,d).

We complemented the behavioural tests with electrophysiological studies of nociceptive input into wide dynamic range (WDR) neurons in the spinal cord of mice, adapting a published method^30^ (Fig. 4e,f). In these experiments, polysynaptic input from peripheral neurons drives activity of deep dorsal horn neurons. Action potentials elicited in second-order neurons of the dorsal horn can then be used to quantify innocuous and noxious input into the central nervous system. Such data are useful in that they complement behavioural studies with numerical data on electrical activity. Baseline evoked activity to mechanical and thermal stimulation was lower in Na_v_1.7 KOs than in littermates and naloxone enhanced WDR firing in KO but not in littermate mice in response to mechanical and thermal stimuli (Fig. 4e,f). Innocuous input evoked by brush stimulation was little affected (Fig. 4f). Both male and female null mutant Na_v_1.7 mice showed enhanced dorsal horn firing to noxious mechanical and thermal input in the presence of naloxone, consistent with the behavioural studies of naloxone action (Fig. 4e,f). Thus, opioid action in Na_v_1.7-null mutant mice appears target nociceptive input from the peripheral nervous system, consistent with expression of μ-opioid receptors on small nociceptive afferents, as well as the cellular specificity of Na_v_1.7 deletion in the advillin-Cre Na_v_1.7 KO mice.

*Ceacam10* was the most downregulated transcript in the Na_v_1.7 microarray analysis and thus we examined the effects of gene deletion on pain behaviour as well. *Ceacam10*-null mutant mice have a limited analgesic phenotype to thermal stimuli in the Hargreaves test and the heterozygous null mutant mice also show partial analgesia (Fig. 4g). Naloxone does not reverse the analgesic phenotype of *Ceacam10*-null mice (Fig. 4g), suggesting that a non-opioid-dependent mechanism may also make a small contribution to the CIP analgesic phenotype associated with loss of Na_v_1.7 expression.

**Figure 5:**
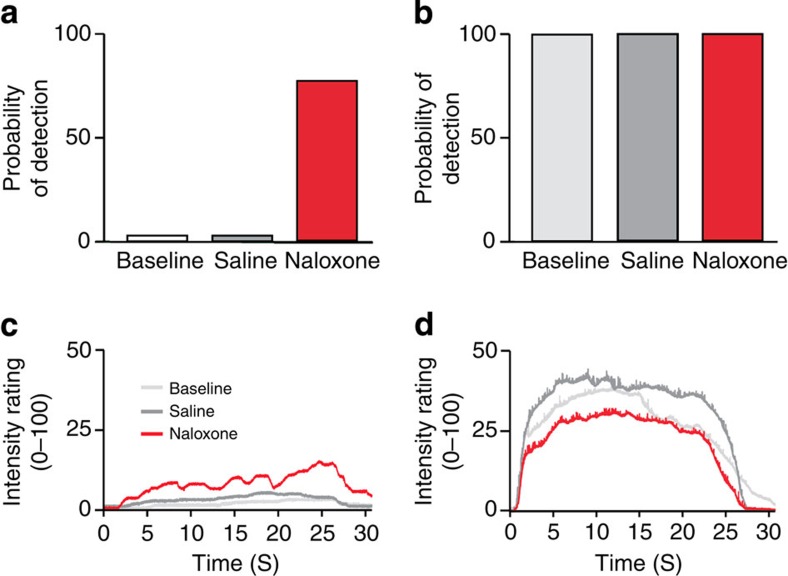
Naloxone dramatically reverses the analgesia associated with Na_v_1.7-null mutations in a CIP individual. Perception of phasic pain. The probability of detecting 9-ms radiant heat pulses was assessed in a Na_v_1.7-null patient (**a**) and in three age-matched healthy controls (**b**). Neodymium:yttrium-aluminum-perovskite (Nd:YAP) laser pulses (El.En, Italy) selectively stimulate intra-epidermal free-nerve endings, thus providing a pure nociceptive input without touch. The human Na_v_1.7 null did not detect any stimuli in baseline and saline conditions. The probability of detecting the stimulus dramatically increased to 80% of stimuli detected during the infusion of 12 mg naloxone, almost reaching the detection levels of matched healthy controls. (**c**,**d**) Tonic pain was elicited by 25 s laser stimuli, whereas the participants rated online the intensity of the heat sensation on a visual analogue scale (0=no sensation, 100=worst pain imaginable) throughout the laser application. Again, naloxone strongly enhanced tonic pain sensations in the Na_v_1.7-null patient (**c**) throughout the time course of the stimulation without effect in the control subjects (**d**).

Finally, we examined the effect of naloxone on pain thresholds in a rare human Na_v_1.7-null CIP individual described and genotyped in detail in ref. 31. Consistent with the KO mouse data, there was a dramatic reversal of analgesia on infusion of naloxone. The ability of the Na_v_1.7-null CIP individual to detect a single noxious heat stimulus in the presence of naloxone rose from 0 to 80% (Fig. 5a,b). In normal conditions, the Na_v_1.7-null subject was completely unaware of the stimulus (Fig. 5a). Ongoing thermal pain could also be detected by the Na_v_1.7-null individual only in the presence of naloxone (Fig. 5c). As well as a response to specific brief noxious stimuli, the CIP patient mentioned general pain in one leg that had suffered several fractures. It would be useful to examine larger numbers of the extremely rare CIP individuals to extend these studies. Nonetheless, the mechanistic studies in mice seem to also explain aspects of the human Na_v_1.7-null CIP phenotype.

In summary, the loss of Na_v_1.7 expression in *SCN9A*-null mice and humans leads to the upregulation of an endogenous opioid system that contributes substantially to the CIP pain-free state through inhibition of nociceptive sensory neuron input into the spinal cord. The poor efficacy of selective Na_v_1.7 blockers may be explained in part by this genetic analysis. If a complete loss of Na_v_1.7-mediated sodium flux is required to upregulate the opioid system, then even potent antagonists are likely to fail. We found that TTX levels >10-fold higher than the IC_50_ for Na_v_1.7 were required to partially recapitulate the upregulation of *Penk* mRNA found in Na_v_1.7-null mutant sensory neurons, suggesting that a complete block of sodium flux through Na_v_1.7 is necessary for *Penk* mRNA upregulation. Ongoing opioid-dependent analgesia throughout the lifetime of the CIP individual is consistent with recent studies on tonic opioid receptor activation in mice^32^. A dramatic synergy in analgesia with co-administration of sodium channel blockers and opioids has already been noted^33^.

Lowered levels of intracellular sodium have been causally linked with increased *Penk* mRNA levels in the present mouse study, consistent with a possible role for sodium as a second messenger. Interestingly, clinical studies have reported increased levels of circulating met-enkephalin associated with lowered systemic sodium levels in idiopathic oedema^34^. The sodium and tonicity-regulated transcription factor NFAT5 is present in both nociceptive and non-nociceptive sensory neurons, and salt kinases such as SGK1 that regulate NFAT5 are also found in sensory neurons^28^. Several copies of the NFAT5 consensus sequence 5′-TGGAAANYNY-3′ are also found upstream of the human *PENK* gene, so that the molecular apparatus defined in the kidney and heart for salt or tonicity regulation of gene expression are also present in DRG sensory neurons^22^,^23^. This is a potential mechanism for sodium regulation of opioid peptide expression.

The A-type G protein-coupled receptors that mediate the actions of opioid peptides are also regulated by a sodium-binding pocket that is linked to agonist binding and G protein coupled receptor activity^35^. Thus, sodium downregulates both the expression of opioid peptides and the activity of their cognate receptors. A possible link between the electrical activity of the voltage-gated sodium channel Na_v_1.7 and the expression and activity of opioid peptides on their cognate receptors is suggested by the present study. Sodium channels have been implicated in a range of physiological functions other than action potential propagation, including hormone secretion and tumour metastases^4^,^36^, although the molecular mechanisms underlying these events are incompletely understood. It is possible that sodium plays a regulatory role in some of these events. The present observations provide a molecular rationale for the use of selective Na_v_1.7 channel blockers that are likely to have few side effects, in combination with presently available opioid drugs (or enkephalinase inhibitors) for the treatment of a large number of chronic pain conditions.

## Methods

### Animals

Both female and male mice aged 8–12 weeks were kept on a 12-h light/dark cycle and provided with food and water *ad libitum*. Conditional Na_v_1.7 KO mice were generated by crossing floxed (SCN9A) Na_v_1.7 mice with Advillin-Cre mice^7^,^16^. Ceacam10 global null mutant mice on a BALBc background were supplied by EMMA (EM:00196 BALB/c-Ceacam10). All experiments were performed with approval from the United Kingdom Home Office according to guidelines set by personal and project licenses, as well as guidelines of the Committee for Research and Ethical Issues of IASP.

### Microarrays

Microarray data were obtained using the Affymetrix GeneChip Mouse Genome 430 2.0 Array. Dorsal root ganglia from all spinal levels of littermate control and Na_v_1.7-, Na_v_1.8- and Na_v_1.9-null mutants (*n*=3) were extracted with RNAeasy kits and triplicate replicates were analysed on the gene arrays. Microarray data were deposited at Gene Expression Omnibus array express for public use with reference number E-GEOD-61373.

### DRG cultures

DRG from all spinal levels were harvested and dissociated using a standard enzymatic protocol previously described^18^. Following incubation in Dispase (Gibco)/collagenase (type XI, Sigma, UK) for 40 min, DRGs were mechanically triturated with three fire-polished glass pipettes of decreasing diameters. DRG cells were centrifuged and re-suspended in DMEM medium supplemented with 10% fetal bovine serum, 1% Glutamax and 125 ng ml^−1^ NGF (Sigma). Dissociated neurons were plated on poly-L-lysine and Laminin-coated 35-mm plastic dishes (Nunc, Denmark). Cultured DRG neurons were incubated with monensin (Sigma; in ethanol absolute), TTX (Sigma; in extracellular solution) or ionomycin (Molecular Probes, Life Technologies; in dimethyl sulfoxide) at concentrations described in the figure legend before RNA extraction and quantification. For each experiment, control DRG neurons were treated with appropriate vehicle.

### RNA quantification

DRG from lumbar segments L4, L5 and L6 were dissected and pooled. RNA was extracted using TRIzol Reagent (Invitrogen) according to the manufacturer's instructions. In the case of DRG cultures, DRG were first dissociated and incubated with the drugs for a given time before collection. Reverse transcription was performed using iScript Reverse Transcription Supermix for reverse transcriptase–qPCR following the supplied protocol by Bio-Rad. Complementary DNA amplification was performed in triplicate, using SsoAdvanced Universal SYBR Green Supermix (Bio-Rad). The following primers were used (5′–3′): *Gapdh* forward TGCGACTTCAACAGCAACTC and reverse CTTGCTCAGTGTCCTTGCTG; *Penk* forward TTCAGCAGATCGGAGGAGTTG and reverse GAAGCGAACGGAGGAGAGAT; and *Ceacam10* forward AGAATTAAATGCAAGGGGGC and reverse ATGAGGGAGCCTACGCACTA. DNA amplification was quantified with a Bio-Rad CFX Connect Real-Time PCR Detection System thermocycler. The expression level of target genes was normalized to housekeeping gene mRNA (*Gapdh*). Fold changes were determined using the 2^−ΔΔCt^ equation in which wild-type littermate or vehicle-treated cultured DRG cDNA samples were designated as the calibrator. The data presented are given as the mean of the fold changes. Student's *t*-test or one-way analysis of variance followed by Dunnett's post test were respectively used to compare expression levels between two groups or three and more groups.

### Immunohistochemistry

Mice were anaesthetized with intraperitoneal pentobarbital and transcardially perfused with PBS (0.01% Heparin) and ice-cold paraformaldehyde (4% in PBS). Lumbar spinal cord fragments were dissected and post fixed for at least 1 h in paraformaldehyde and transferred to a sucrose solution (0.3 M) for cryoprotection. Tissues were embedded in optical coherence tomography mounting medium, frozen in liquid nitrogen and stored at −80 °C. Free-floating sections (30 μm) were blocked 1 h in PBS with 10% goat serum (Sigma) and 0.1% Triton X-100, followed by overnight incubation at 4 °C in Rabbit anti-met-enkephalin primary antibody (1:500 in PBS with 3% goat serum and 0.1% Triton X-100, AB5026, Millipore). Co-staining with Isolectin B4 biotin conjugate (IB4, Sigma) was used to label spinal cord lamina II. Corresponding monoclonal goat anti-rabbit met-enkephalin (1:1,000 Alexa Fluor 488, A11017, Life Technology, UK) or anti-biotin (1:1,000 Streptavidin Alexa Fluor 568 conjugate, S-11226, Life Technology) secondary antibodies were used. Spinal cord slices were imaged using a Leica TCS SP8 spectral confocal microscope (Leica Microsystems, Germany) using laser lines 488 nm (Met-enk) and 552 nm (IB4) at an acquisition frequency of 200 Hz (1,024 × 1,024 format). The laser power and acquisition frequency was kept constant between all respective samples. The relative fluorescence for met-enkephalin was calculated by taking the mean fluorescence of the entire dorsal horn (indicated by IB4 staining) and subtracting background fluorescence (LAS AF 3 analysis software). The relative fluorescence for each animal was taken as an average of at least three spinal sections. Three animals were used per group.

### Mouse behaviour

All behavioural experiments were performed by an experimenter blind to both genotype and treatment. Mechanical nociceptive thresholds were measured using the Randall-Selitto test that applies pressure to the tail with a 3-mm^2^ blunt conical probe using a 500 g cutoff. Thermal nociceptive thresholds were determined by measuring paw-withdrawal latency using the Hargreaves apparatus at a ramp of 2.25 °C s^−1^ with a 20-s cutoff. Naloxone hydrochloride dihydrate (2 mg kg^−1^ intraperitoneally, Sigma) was administered 30 min before behavioural assessment. Statistical differences between KOs and littermates, as well as the effects of naloxone in each genotype group, were determined using a two-way analysis of variance.

### *In vivo* electrophysiology

Electrophysiological recordings were performed by an experimenter blind to genotype. Mice were anaesthetized with isofluorane (4%; 66% N_2_O and 33% O_2_) and secured in a stereotaxic frame. Anaesthesia was reduced and maintained at 1.5% isoflurane for the remaining duration of the experiment. A laminectomy was performed to expose L3–L5 segments of the spinal cord and extracellular recordings were made from WDR neurons in the deep dorsal horn (lamina III–V, 200–600 μm) using parylene-coated tungsten electrodes (A-M Systems, USA) in Na_v_1.7 KO mice (*n*=17 neurons) and littermate controls (*n*=24 neurons). Mechanical and thermal stimuli were applied to the peripheral receptive field of spinal neurons on the hindpaw glabrous skin and the evoked activity of neurons was visualized on an oscilloscope and discriminated on a spike amplitude and waveform basis using a CED 1401 interface coupled to Spike 2 software (Cambridge Electronic Design, UK). Natural stimuli (non-noxious 8 g von Frey and noxious prod 100 g cm^−2^ mechanical stimulation; thermal water jet 40, 45 °C) were applied in ascending order of intensity to receptive fields for 10 s and the total number of evoked spikes recorded. Following baseline recordings of evoked activity of deep dorsal horn neurons, naloxone was administered (2 mg kg^−1^ subcutaneously, Sigma) and evoked activity to natural stimuli was characterized again. Statistical significance for differences between littermates and KOs was determined using a *t*-test; effects of naloxone on littermates and KOs were determined using paired t-tests.

### Human behaviour

All participants gave written informed consent. The study was approved by the UCL Research Ethics Committee. The CIP patient was a 39-year-old woman. Healthy controls were age matched (*N*=3, mean age±s.d.: 38.7±3.2). Perception of phasic and tonic radiant heat was assessed at baseline and during intravenous administration of saline or naloxone (12 mg), in a randomized order. Psychophysical assessment was carried out by an experimenter blind to the pharmacological condition. Radiant heat was generated by an infrared neodymium:yttrium-aluminum-perovskite laser with a wavelength of 1.34 μm (Electronical Engineering, Italy). This method was used to selectively activate free nerve endings in the superficial layers of the hairy skin^37^,^38^. The laser fluency was 0.6 J mm^−2^ and the duration of each laser pulse was 9 ms. After each of the ten pulses delivered at random intervals (15–40 s) to the forearm, participants were asked to verbally report whether they detected any stimulus. Tonic radiant heat was generated by a CO_2_ laser, whose power is regulated using a feedback control based on an online measurement of skin temperature at the site of stimulation (Laser Stimulation Device, SIFEC, Belgium). On each trial, tonic radiant heat was delivered to the forearm for 25 s, at one of three possible temperatures: 42, 45 and 48 °C (ref. 37). Participants were asked to rate the intensity of the thermal sensation on a visual analogue scale throughout the trial (0=no sensation, 100=worst pain imaginable). Three trials per stimulus temperature were given on each session (baseline, saline and naloxone) in a randomized order.

## Additional information

**Accession codes:** Microarray data of DRG tissues extracted from Na_v_1.7 KO, Na_v_1.8 KO and Na_v_1.9 KO mice are available in the ArrayExpress and Gene Expression Omnibus (GEO) databases, accession code GSE61373.

**How to cite this article:** Minett, M. S. *et al*. Endogenous opioids contribute to insensitivity to pain in humans and mice lacking sodium channel Na_v_1.7. *Nat. Commun*. 6:8967 doi: 10.1038/ncomms9967 (2015).

## Supplementary Material

Supplementary Information Supplementary Tables 1-2.
