# Supplementary material for: Endogenous opioids contribute to insensitivity to pain in humans and mice lacking sodium channel Nav1.7
Source: Nat Commun. 2015 Dec 4;6:8967. doi: 10.1038/ncomms9967 (PMC4686868; doi:10.1038/ncomms9967)
Supplement: Supplementary Information — Supplementary Tables 1-2. [file ncomms9967-s1.pdf]

Supplementary Table 1 Genes mis-expressed in both Nav1.7 and Nav1.8 null mutant DRG

Gene	Fold-change Nav1.7	Fold-change Nav1.8
Ceacam10	- 18.94	- 2.63
Smr2	+1.96	-1.78
Lrrc31	-4.37	-1.66

Three transcripts are common to the altered transcriptome in Nav1.7 and Nav1.8 null mutant dorsal root ganglia, although *Smr2* is upregulated in the absence of Nav1.7 but downregulated in the absence of Nav1.8.

Supplementary Table 2 Genes mis-expressed in both Nav1.7 and Nav1.9 null mutant DRG

Gene	Fold change Nav1.7	fold change Nav1.9
Ptger1	+2.68	-1.92
Sox11	+1.57	-1.63
Lrat	-1.57	-1.71
Npy1r	-1.8	-1.68
Jun	+1.94	-1.52
Mbp	-1.52	+1.52
Msi2	-1.52	+1.6

Several genes are dysregulated in both Nav1.7 and Nav1.9 null mutant mouse DRG , but only 2 (*Npy1r* and *Lrat*) are jointly downregulated.
